# Echocardiographic Predictors of Postoperative Atrial Fibrillation After Cardiac Surgery: Assessing Atrial Mechanics for Risk Stratification

**DOI:** 10.3390/jcdd12040160

**Published:** 2025-04-17

**Authors:** Velimir Perić, Mlađan Golubović, Marija Stošić, Dragan Milić, Lela Lazović, Dalibor Stojanović, Milan Lazarević, Dejan Marković, Dragana Unić-Stojanović

**Affiliations:** 1 Clinic for Cardiac Surgery, University Clinical Center Nis, 18000 Nis, Serbia; mladjangolubovic@gmail.com (M.G.); marija91nis@gmail.com (M.S.); drdraganmilic@gmail.com (D.M.); adalazovic@gmail.com (L.L.); dalibor.stojanovic08@gmail.com (D.S.); 2 Faculty of Medicine, University of Nis, 18000 Nis, Serbia; dr_m.lazarevic@hotmail.com; 3 Institute Niska Banja, 18000 Nis, Serbia; 4 Center for Anesthesiology and Reanimatology, University Clinical Center of Serbia, 11000 Belgrade, Serbia; drdejanmarkovic@gmail.com; 5 Medical Faculty, University of Belgrade, 11000 Belgrade, Serbia; dragana.unic@gmail.com; 6 Institute for Cardiovascular Diseases Dedinje, 18000 Belgrade, Serbia

**Keywords:** postoperative atrial fibrillation, echocardiography, atrial function, right atrium, left atrium, POAF prediction, risk stratification

## Abstract

Postoperative atrial fibrillation (POAF) is a common complication after cardiac surgery, increasing morbidity and healthcare costs. This study aimed to identify echocardiographic predictors of POAF to improve risk stratification. A total of 131 patients undergoing cardiac surgery were analyzed and divided into two groups based on POAF occurrence. Echocardiographic analysis showed that patients with POAF had larger left and right atrial dimensions and impaired atrial function. Prolonged total atrial conduction time (TACT), reduced atrial emptying volumes, and contractile function were more common in the POAF group. Univariable analysis identified LAEF (χ^2^ = 71.8, *p* < 0.001), LAKE (χ^2^ = 70.1, *p* < 0.001), RATEF (χ^2^ = 65.7, *p* < 0.001), and RAAEF (χ^2^ = 66.8, *p* < 0.001) as significant predictors of POAF, each with an area under the curve (AUC) greater than 0.89. In multivariable analysis, LAKE (OR = 0.27, *p* < 0.001), hypertension (OR = 11.87, *p* = 0.035), left ventricular ejection fraction (OR = 1.08, *p* = 0.020), and peripheral vascular disease (OR = 40.28, *p* = 0.002) were independent predictors. The final model showed a significant discriminatory ability (AUC = 0.94). LAKE and clinical factors remained independent predictors after adjustment.

## 1. Introduction

Atrial fibrillation is the most frequent arrhythmia in both the general population and postoperative settings. According to the consensus definition implemented in the 2020 guidelines of the European Society of Cardiology (ESC) and the European Association for Cardio-Thoracic Surgery (EACTS), atrial fibrillation is characterized by uncoordinated atrial electrical activity, with confirmation requiring either an electrocardiogram (ECG) trace lasting at least 30 s or characteristic atrial fibrillation features present in all 12 electrocardiogram leads [1].

Postoperative atrial fibrillation (POAF) is a newly occurring arrhythmia with the highest incidence following cardiac surgical procedures, largely due to the high frequency of cardiovascular comorbidities, risk factors, and myocardial injury specific to these surgeries. POAF is the most frequent major adverse cardiac event (MACE) and one of the most common postoperative complications, occurring in up to 65% of cardiac surgery patients [2]. Patients who develop POAF have an eightfold increased risk of recurrent or permanent atrial fibrillation [3], and those who develop POAF within three years of coronary artery bypass grafting (CABG) have a threefold increased risk of all-cause mortality and a fourfold higher incidence of stroke than those without POAF [4]. Additionally, POAF is linked to other severe postoperative complications, such as acute respiratory distress syndrome, pneumonia, septicemia, thromboembolism, sudden cardiac death, reoperation, and internal bleeding [3]. The financial burden of POAF is significant, prolonging hospitalization by an average of five days and increasing treatment costs by more than USD 10,000 per patient, contributing to over USD 2 billion in annual healthcare expenditures in the United States, according to the American Heart Association [5].

The clinical application of risk scores offers an opportunity to enhance patient outcomes by improving preoperative evaluation and preparation through additional diagnostic or therapeutic measures, optimizing perioperative and postoperative monitoring to assess and mitigate POAF development, and reducing the risk of long-term atrial fibrillation and its complications through tailored interventions for high-risk patients.

Echocardiography plays a vital role in assessing and managing POAF, providing detailed visualization of cardiac structures and functions to identify risk factors such as left atrial enlargement, right atrial dilation, diastolic dysfunction, and valvular abnormalities [6]. Doppler and tissue imaging techniques can evaluate atrial strain, left ventricular filling pressures, and atrial electromechanical delay, all of which are associated with an increased risk of atrial arrhythmias [7]. Moreover, echocardiography is essential for detecting pericardial effusion, myocardial ischemia, and ventricular dysfunction, all of which may exacerbate POAF [8]. Transesophageal echocardiography (TEE) is particularly useful for ruling out atrial thrombi before cardioversion in patients with prolonged or recurrent atrial fibrillation [9]. By guiding risk stratification, anticoagulation decisions, and treatment strategies, echocardiography remains an indispensable tool in optimizing postoperative outcomes [10].

As part of this research, we investigated the predictive potential of echocardiographic parameters for POAF development, specifically examining left and right atrial diameters, left ventricular systolic and diastolic function, and pulmonary artery pressure. Special attention was given to maximal atrial volumes and their indexing to body surface area, as these parameters are potential markers of atrial remodeling and predisposition to atrial arrhythmias.

Given the pathophysiological complexity of POAF, our study aimed to determine whether echocardiographic markers, particularly those associated with atrial structure and function, can improve risk stratification and clinical decision-making in the perioperative setting. Identifying high-risk patients before surgery could allow for targeted preventive strategies, including optimized medical therapy, rhythm control interventions, and enhanced postoperative monitoring.

In addition to univariable analysis, we performed multivariable logistic regression to identify independent predictors of POAF after adjusting for clinical factors such as age, hypertension, left ventricular ejection fraction, and peripheral vascular disease. This approach allowed us to determine the relative contribution of echocardiographic markers while accounting for the influence of established clinical risk factors. Our findings contribute to the growing body of evidence supporting echocardiography’s role not only in diagnosing and managing atrial fibrillation but also in predicting and preventing POAF in surgical patients. Incorporating these echocardiographic predictors into routine preoperative assessments may reduce POAF incidence, improve patient outcomes, and lower healthcare costs associated with prolonged hospital stays and postoperative complications.

## 2. Materials and Methods

### 2.1. Study Design and Patient Selection

This research was conducted as a prospective observational monocentric study that included 131 patients undergoing elective or emergency surgical myocardial revascularization, aortic valve replacement with a mechanical prosthesis, and combined procedures during a 5-month period, starting in May 2024 at the Clinic for Cardiac Surgery, University Clinical Center in Nis, Serbia. This research was performed in accordance with the Helsinki Declaration principles and approved by the Ethics Committee of the Faculty of Medicine, University of Nis, and the Ethics Committee of the University Clinical Center Nis, Nis, Serbia. A higher POAF incidence after mitral and tricuspid surgery procedures and a small number of such procedures during the study period were reasons for their exclusion. Furthermore, we considered the pathophysiological complementarity of aortic stenosis and coronary artery disease, as these conditions share numerous risk factors and frequently coexist [11].

The primary outcome was the occurrence of new-onset POAF during the in-hospital period. POAF was defined according to the consensus definition implemented in the 2020 guidelines of the European Society of Cardiology (ESC) and the European Association for Cardio-Thoracic Surgery (EACTS) [1]. Essential exclusion criteria were as follows: age under 18 years, previous atrial fibrillation and/or flutter, supraventricular rhythm disorders, presence of a permanent pacemaker, previous radiofrequency ablation or Cox maze procedure, and urgent cardiac surgery.

All patients in the study were under intensive monitoring for a minimum of seven days, which included continuous ECG monitoring and daily analysis of morning 12-channel ECGs, as well as its mandatory use as evidence of heart rhythm changes.

Preoperatively, all patients underwent a detailed review of medical history; physical examination; and echocardiographic, ECG, and laboratory evaluation. The patients were assessed for anesthetic risk using the American Society of Anesthesiologists (ASA) classification [12], while the New York Heart Association (NYHA) classification was used to assess the severity of heart failure symptoms [13]. POAF, CHA2DS2-VASc, and HATCH risk scores and the Atrial Fibrillation Risk Index (AFRI) were quantified preoperatively to test their predictive characteristics.

### 2.2. Preoperative Echocardiographic Atrial Function Assessment

All patients were examined preoperatively by an anesthesiologist, cardiac surgeon, and cardiologist. The day before the operation, all patients underwent a detailed echocardiographic examination by a cardiologist according to the recommendations of the American Society of Echocardiography (ASE) and European Association of Cardiovascular Imaging (EACVI) using a GE Vivid 6 (GE Healthcare Milwaukee, West Milwaukee, WI, USA) ultrasound machine [14]. Preoperative transthoracic echocardiographic evaluation included two-dimensional (2D), M-mode, pulsed-wave, color, and tissue Doppler imaging. Atrial volumes are measured at different time points of the cardiac cycle: maximal atrial volumes at the end of the T wave on the electrocardiogram, just before opening of the corresponding atrioventricular valve; minimal atrial volumes at QRS complex, immediately upon closing of the corresponding atrioventricular valve; and the atrial volumes in front of atrial contraction (pre-A) at the beginning of the P wave. The biplane area–length method was used after obtaining adequate apical four-chamber and two-chamber views. All measured volumes were indexed in relation to body surface area. Left and right atrial functions were additionally examined using the following parameters:

(1) Contractile function: A-wave velocities, left and right atrial kinetic energy (LAKE; RAKE), left and right atrial ejection force (LAEF, RAEF), and left and right atrial active emptying fractions (LAAEF; RAAEF).

(2) Conduit function: left and right atrial passive emptying fractions (LAPEF; RAPEF).

(3) Reservoir function: left and right atrial total emptying fractions (LATEF; RATEF).

(4) Electrical function: total atrial conduction time (TACT).

Detailed explanations and calculation procedures for the listed parameters are found in Table 1. Strain and strain rate imaging were not utilized in this study.

### 2.3. Operative Procedure Characteristics

All procedures were performed under general balanced endotracheal anesthesia. Median sternotomy was the surgical approach for all patients. Patients who underwent cardiopulmonary bypass received conventional cardioprotection with cold, crystalloid, antegrade, and intermittent cardioplegia. Surgical revascularization of the myocardium was performed using autologous arterial (internal thoracic artery) and/or venous grafts (deep saphenous vein) with or without cardiopulmonary bypass. The intraoperative cell salvage technique was implemented for all patients.

### 2.4. Statistical Analysis

All statistical analyses were performed using SPSS 21.0 (IBM Corp., Chicago, IL, USA). Categorical variables were summarized as frequencies and percentages, while continuous variables were expressed as means with standard deviations or medians with interquartile ranges, depending on data distribution. Group comparisons for continuous variables were performed using Student’s *t*-test for normally distributed data and the Mann–Whitney U test for non-normally distributed data. Categorical variables were compared using Fisher’s exact test. Univariable binary logistic regression was used to identify predictors of POAF, with results reported as odds ratios (ORs) and 95% confidence intervals (CIs). To assess the predictive accuracy of individual echocardiographic parameters (LAEF, LAKE, RATEF, and RAAEF), receiver operating characteristic (ROC) curve analysis was performed, and the area under the curve (AUC) was calculated for each parameter. To identify independent predictors of POAF, a multivariable Cox proportional hazards model was constructed, incorporating significant echocardiographic predictors from the univariable analysis and clinically relevant factors such as age, hypertension, left ventricular ejection fraction, peripheral vascular disease, and chronic obstructive pulmonary disease (COPD). A backward stepwise elimination approach was applied to retain the most parsimonious model. Multicollinearity was evaluated using the variance inflation factor (VIF), and predictors with a VIF > 5 were excluded from the final model. Adjusted hazard ratios (HRs) with 95% confidence intervals were reported for the significant predictors.

## 3. Results

A total of 131 patients were included in this analysis, divided into two groups: those who developed POAF and those who did not. In total, 100 patients (76.3%) underwent surgical myocardial revascularization (with 88% performed under cardiopulmonary bypass), 19 patients (14.5%) underwent aortic valve replacement, and 12 patients (9.2%) underwent combined aortic valve and coronary surgery. Of the 131 patients, 84 (64%) did not develop POAF, while 47 (36%) did. Significant differences were observed between the two groups in terms of demographic, clinical, and echocardiographic characteristics (Table 2).

Patients who developed POAF were significantly older than those who did not (68.7 ± 7.64 vs. 63.86 ± 7.91 years, *p* = 0.001). Gender distribution showed no statistically significant difference, with 76.6% of POAF patients and 75.0% of non-POAF patients being male (*p* = 1.000). Other anthropometric measures showed no significant differences between groups, including weight, height, body surface area (BSA), and body mass index (BMI).

The CHA_2_DS_2_-VASc score, a well-established predictor of atrial fibrillation and thromboembolic risk, was higher in the POAF group than in the non-POAF group, though this difference did not reach statistical significance (2.91 ± 1.02 vs. 2.56 ± 1.21, *p* = 0.076). The POAF score, which specifically estimates the risk of atrial fibrillation following cardiac surgery, was significantly higher in the POAF group (2.13 ± 1.15 vs. 1.54 ± 1.10, *p* = 0.005), suggesting its potential utility in risk stratification. Neither the HATCH score (1.79 ± 0.88 vs. 1.56 ± 1.09, *p* = 0.197) nor the AFRI (2.02 ± 0.94 vs. 1.74 ± 0.78, *p* = 0.084) showed an association with POAF, nor did the ASA (χ^2^ = 0.19, *p* = 0.663) or NYHA (χ^2^ = 6.82, *p* = 0.078) classification.

ECG parameters revealed a significantly longer PR (Q) interval in the POAF group than in non-POAF patients (174.28 ± 22.23 vs. 164.23 ± 27.89 ms, *p* = 0.026), indicating possible alterations in atrioventricular conduction associated with the development of atrial fibrillation.

Among cardiovascular risk factors, smoking was significantly more prevalent in the POAF group than in the non-POAF group (55.32% vs. 33.33%, *p* = 0.02), reinforcing its role as a modifiable risk factor for postoperative arrhythmias. While the presence of chronic obstructive pulmonary disease (COPD) was higher in the POAF group (31.91% vs. 11.9%), this difference did not reach statistical significance (*p* = 0.063), though the trend suggests a potential relationship that warrants further investigation.

Regarding medication use, patients in the POAF group received a higher beta-blocker dose than those in the non-POAF group (8.16 ± 17.53 vs. 3.05 ± 2.40 mg, *p* = 0.052), though this result did not achieve statistical significance. The use of angiotensin-converting enzyme inhibitors (ACE inhibitors), calcium channel blockers, statins, antiplatelet therapy (aspirin and P2Y12 inhibitors), anticoagulation therapy (oral anticoagulants), and amiodarone did not differ significantly between groups. Conversions to equivalent drug dosages were made using the GlobalRPh website [15].

Notably, the presence of peripheral vascular disease (PVD) was significantly more common in the POAF group (17.02% vs. 7.14%, *p* = 0.01), suggesting underlying systemic atherosclerosis and endothelial dysfunction that could predispose patients to increased atrial arrhythmogenicity.

These findings highlight age, smoking, prolonged PR (Q) interval, an increased POAF score, and the presence of PVD as significant predictors of POAF in this cohort. The potential contribution of COPD and beta-blocker dosage to POAF risk warrants further study.

Echocardiographic parameters revealed significant differences between patients who developed postoperative atrial fibrillation (POAF) and those who did not (Table 3). Notably, total atrial conduction time (TACT) was significantly prolonged in the POAF group compared with the non-POAF group (141.93 ± 15.23 vs. 103.59 ± 7.73 ms, *p* < 0.001), suggesting a substantial delay in atrial electrical conduction associated with POAF development.

Right atrial parameters demonstrated marked differences between the two groups. Patients with POAF exhibited significantly larger maximal right atrial volume (maxRAV) (58.89 ± 10.61 vs. 46.03 ± 11.87 mL, *p* < 0.001) and indexed maximal right atrial volume (maxRAVi) (30.52 ± 5.31 vs. 24.03 ± 6.21 mL/m^2^, *p* < 0.001), indicating greater right atrial enlargement in this population. Similarly, minimal right atrial volume (minRAV) and its indexed value (minRAVi) were significantly higher in the POAF group (42.48 ± 10.26 vs. 23.94 ± 7.06 mL, *p* < 0.001 and 22.00 ± 5.12 vs. 12.50 ± 3.71 mL/m^2^, *p* < 0.001, respectively), reflecting increased right atrial volume retention.

Right atrial volume just before atrial contraction (pre-atrial contraction RAV-pacRAV) and its indexed value (pacRAVi) were also significantly larger in patients with POAF than in those without (47.39 ± 9.88 vs. 33.41 ± 8.29 mL, *p* < 0.001 and 24.57 ± 4.98 vs. 17.48 ± 4.57 mL/m^2^, *p* < 0.001, respectively). By contrast, the tricuspid annular orifice area was significantly smaller in the POAF group (7.19 ± 0.31 vs. 8.13 ± 0.34 cm^2^, *p* < 0.001), which may indicate functional right heart alterations predisposed to atrial fibrillation.

Right ventricular functional assessment showed that tricuspid annular plane systolic excursion (TAPSE) was significantly reduced in POAF patients (16.78 ± 4.17 vs. 20.09 ± 3.09 mm, *p* < 0.001), whereas mitral annular plane systolic excursion (MAPSE) did not significantly differ between the two groups (*p* = 0.122). Analysis of tricuspid inflow (TI) velocities revealed that late diastolic velocity (TI A-wave) was significantly lower in POAF patients (0.31 ± 0.15 vs. 0.47 ± 0.08 m/s, *p* < 0.001), while the early-to-late diastolic velocity ratio (TI E/A) was significantly increased (1.91 ± 0.64 vs. 1.17 ± 0.30, *p* < 0.001).

Left atrial mechanical function was significantly impaired in POAF patients. Left atrial total emptying volume (LATEV) and its indexed value (LATEVi) were significantly lower in the POAF group (21.17 ± 8.20 vs. 29.24 ± 6.60 mL, *p* < 0.001 and 10.68 ± 4.30 vs. 14.98 ± 4.38 mL/m^2^, *p* < 0.001, respectively), while left atrial total emptying fraction (LATEF) was also markedly reduced (0.28 ± 0.10 vs. 0.44 ± 0.11%, *p* < 0.001).

Left atrial passive emptying volume (LAPEV) and its indexed value (LAPEVi) did not show significant differences between the two groups (*p* = 0.402 and *p* = 0.311, respectively). However, left atrial passive emptying fraction (LAPEF) was significantly lower in POAF patients (0.20 ± 0.09 vs. 0.24 ± 0.09%, *p* = 0.022). Similarly, left atrial active emptying volume (LAAEV) and its indexed value (LAAEVi) were significantly decreased in POAF patients (5.76 ± 2.09 vs. 12.68 ± 5.83 mL, *p* < 0.001 and 2.92 ± 1.15 vs. 6.25 ± 3.43 mL/m^2^, *p* < 0.001, respectively), with a corresponding reduction in left atrial active emptying fraction (LAAEF) (0.10 ± 0.03 vs. 0.26 ± 0.12%, *p* < 0.001).

Key atrial function markers, left atrial ejection force (LAEF), and kinetic energy of the left atrium (LAKE), were significantly lower in POAF patients than in non-POAF patients (0.51 ± 0.65 vs. 1.85 ± 0.94 kdyne, *p* < 0.001 and 0.91 ± 1.36 vs. 5.13 ± 3.40 kdyne·cm, *p* < 0.001, respectively), suggesting severe impairment in left atrial contractile function.

Right atrial total emptying volume (RATEV) and its indexed value (RATEVi) were significantly lower in POAF patients (16.41 ± 5.28 vs. 22.09 ± 8.96 mL, *p* < 0.001 and 8.34 ± 2.96 vs. 10.84 ± 5.33 mL/m^2^, *p* = 0.0007, respectively), with a notable reduction in right atrial total emptying fraction (RATEF) (0.28 ± 0.09 vs. 0.47 ± 0.11%, *p* < 0.001).

Similarly, right atrial passive emptying volume (RAPEV) and its indexed value (RAPEVi) were not significantly different between groups (*p* = 0.320 and *p* = 0.567, respectively). However, right atrial passive emptying fraction (RAPEF) was significantly lower in POAF patients (0.20 ± 0.08 vs. 0.26 ± 0.10%, *p* < 0.001). Right atrial active emptying volume (RAAEV) and its indexed value (RAAEVi) were significantly decreased in the POAF group (4.91 ± 1.03 vs. 9.47 ± 4.82 mL, *p* < 0.001 and 2.51 ± 0.72 vs. 4.68 ± 2.79 mL/m^2^, *p* < 0.001, respectively), with a corresponding reduction in right atrial active emptying fraction (RAAEF) (0.11 ± 0.05 vs. 0.28 ± 0.12%, *p* < 0.001).

Additionally, right atrial ejection force (RAEF) and kinetic energy (RAKE) were significantly lower in POAF patients (0.44 ± 0.47 vs. 0.84 ± 0.34kdyne, *p* < 0.001 and 0.32 ± 0.38 vs. 1.11 ± 0.63 kdyne.cm, *p* < 0.001, respectively), indicating impaired right atrial mechanics.

These findings suggest that patients who develop POAF exhibit significant impairments in both right and left atrial functions, with reduced atrial emptying volumes, lower contractile function, and prolonged conduction times, emphasizing the potential role of echocardiographic parameters in risk stratification and early intervention for POAF.

**Figure 1:**
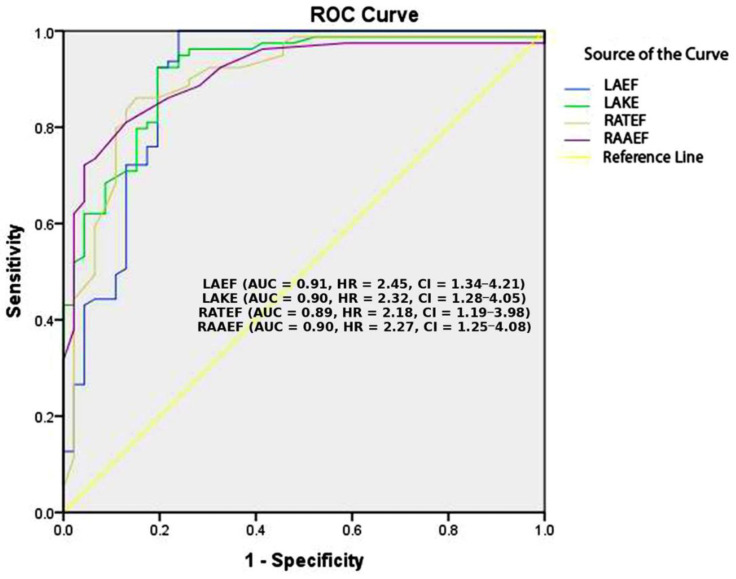
Individual discriminative ability of POAF predictors. LAEF, left atrial ejection force; LAKE, left atrial kinetic energy; RATEF, right atrial total emptying fraction; RAAEF, right atrial active emptying fraction.

The echocardiographic parameters LAEF, LAKE, RATEF, and RAAEF demonstrated strong discriminatory ability in predicting POAF, with an area under the curve (AUC) exceeding 0.89. The Kaplan–Meier graph in Figure 1 illustrates the individual discriminative abilities of these atrial function parameters. Further predictive assessment using binary logistic regression identified these parameters as significant independent predictors of POAF.

Univariable analysis revealed that LAEF was a significant predictor of POAF, with a hazard ratio (HR) and 95% confidence interval (CI) ranging from 0.030 to 0.167 (χ^2^ = 71.842, *p* < 0.001). Similarly, LAKE showed strong predictive value, with an HR (95% CI) of 0.298 to 0.563 (χ^2^ = 70.082, *p* < 0.001). The right atrial total emptying fraction (RATEF) was also significantly associated with POAF (χ^2^ = 65.709, *p* < 0.001), as was the right atrial active emptying fraction (RAAEF) (χ^2^ = 66.80, *p* < 0.001).

**Figure 2:**
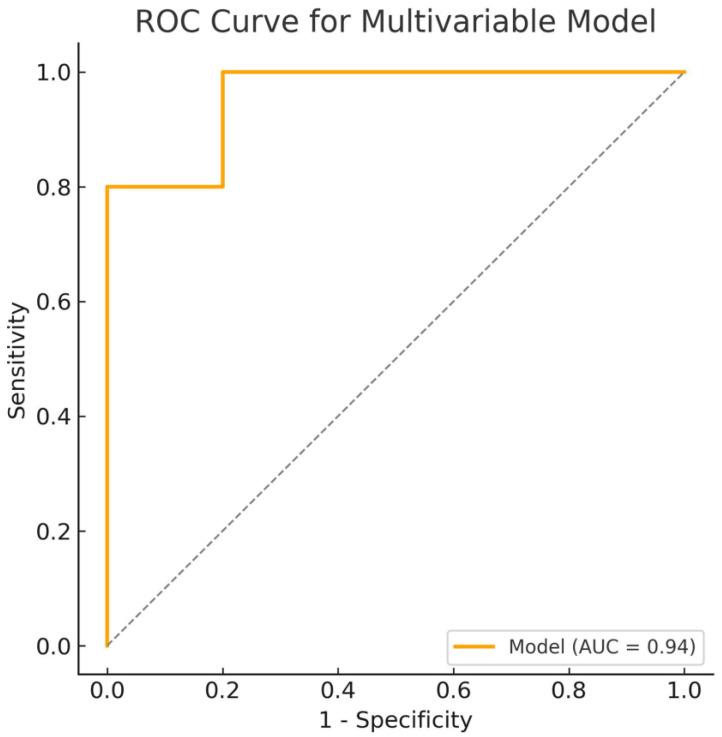
Receiver operating characteristic curve for the multivariable Cox regression model predicting POAF. ROC, receiver operating characteristic; AUC, area under the curve.

In the multivariable Cox regression model (Figure 2.), LAKE (HR = 0.27, 95% CI 0.16–0.46, *p* < 0.001), hypertension (HR = 11.87, 95% CI 1.19–118.25, *p* = 0.035), left ventricular ejection fraction (HR = 1.08, 95% CI 1.01–1.15, *p* = 0.020), and peripheral vascular disease (HR = 40.28, 95% CI 3.81–425.80, *p* = 0.002) emerged as independent predictors of POAF. The final model demonstrated a significant discriminatory ability with an AUC of 0.94 (95% CI 0.86–0.94). The combined model demonstrates superior predictive performance compared with individual predictors, confirming the additive value of combining echocardiographic and clinical parameters in POAF risk stratification. These findings highlight the potential of LAEF, LAKE, RATEF, and RAAEF as valuable echocardiographic markers for risk stratification and the early identification of patients at increased risk for POAF. However, after adjusting for clinical factors, LAKE and clinical parameters (hypertension, left ventricular ejection fraction, and peripheral vascular disease) remained independent predictors of POAF.

## 4. Discussion

Postoperative atrial fibrillation (POAF) remains a frequent and significant complication following cardiac surgery, contributing to increased morbidity, longer hospital stays, and greater healthcare costs. Identifying accurate preoperative predictors of POAF is crucial for risk stratification and developing preventive strategies. Our study demonstrates that LAEF, LAKE, RATEF, and RAAEF can serve as strong echocardiographic predictors of POAF. These findings reinforce the growing recognition that both left and right atrial functions play critical roles in arrhythmogenesis and provide additional insights beyond traditional clinical risk factors.

POAF development is largely influenced by age-related atrial remodeling and conduction abnormalities. In our study, patients who developed POAF were significantly older, consistent with previous findings showing that atrial fibrosis, conduction slowing, and structural remodeling increase with age, predisposing elderly patients to atrial arrhythmias [1]. A prolonged PR (Q) interval in the POAF patients was also observed, suggesting an underlying conduction delay. This supports previous research demonstrating that first-degree atrioventricular block and prolonged PR interval are associated with an increased risk of atrial fibrillation due to electrical remodeling and slowed atrioventricular nodal conduction [2].

Among modifiable risk factors, smoking was significantly more prevalent in the POAF group, reinforcing its role as a contributor to systemic inflammation, oxidative stress, and heightened sympathetic tone, which collectively increases atrial arrhythmogenic potential [16]. Although COPD did not reach statistical significance, its trend toward higher prevalence in POAF patients aligns with previous reports that chronic lung disease exacerbates atrial remodeling through chronic hypoxia, pulmonary hypertension, and right atrial overload [6].

The echocardiographic assessment revealed significant differences in atrial function and conduction between POAF and non-POAF patients. Total atrial conduction time (TACT) was markedly prolonged in POAF patients, highlighting delayed atrial depolarization, increased conduction heterogeneity, and electrical instability, all of which contribute to arrhythmogenesis [7]. Prolonged atrial conduction time has been previously associated with atrial fibrillation recurrence and postoperative arrhythmias, further supporting its role in POAF pathophysiology [9].

Right atrial dilatation is a reliable predictor of mortality and clinical worsening of heart failure, pulmonary hypertension, and COPD. Disturbances in the systolic and diastolic function of the right ventricle are associated with POAF [17]. The morphology and function of the right atrium are affected by pulmonary hypertension and the functions of the right ventricle, left atrium, and left ventricle. Right atrial pressure and/or volume load can trigger fibrosis, that is, atrial remodeling [10,18]. Although the ostia of the pulmonary veins are the most common site of triggered electrical activity in cardiac surgical patients with POAF, the following arrhythmogenic loci belonging to the right atrium have been described: the ostia of the coronary sinus, the upper and lower vena cava, and the atrial appendage [19,20]. Guided by the association of left ventricular function parameters with POAF occurrence and the morphological and functional parameters of the left atrium, as well as their predictive capabilities, we tested the significance of the volume, volume indices, diameter, and fraction of the right atrium for the occurrence of POAF [17]. Both atria share the same embryological origin and cellular structure. There are few studies on the importance of right atrial parameters in evaluating POAF. For example, Aksu et al. distinguished maximal left and right atrial volume indices as independent POAF predictors [20].

Right atrial enlargement and dysfunction were particularly pronounced in our POAF patients. There are data on the association between POAF after coronary surgery and the preoperative electrical and structural substrate of the right atrial myocardium. Maximal and minimal right atrial volumes (maxRAV; minRAV) and their indexed values (maxRAVi; minRAVi) were significantly larger in our POAF patients, suggesting right atrial dilation and impaired contractility. Previous studies have demonstrated that right atrial enlargement is associated with increased atrial pressure, fibrosis, and electrical remodeling, making it an independent predictor of atrial fibrillation [10]. Additionally, the smaller tricuspid annular orifice area observed in our POAF patients suggests an altered right-heart hemodynamic profile, which may contribute to atrial volume overload, mechanical dysfunction, and increased susceptibility to arrhythmias [16].

In parallel, left atrial function was significantly impaired in our POAF patients. Reduced left atrial total emptying volume (LATEV), passive emptying volume (LAPEV), and active emptying volume (LAAEV) were observed, along with corresponding reductions in left atrial total emptying fraction (LATEF), passive emptying fraction (LAPEF), and active emptying fraction (LAAEF), underscoring left atrial dysfunction as a major determinant of POAF. These findings align with studies indicating that impaired atrial reservoir and contractile function predispose patients to atrial fibrillation by promoting electrical and mechanical remodeling [21].

Dilatation of the left atrium is a sign of atrial remodeling and an arrhythmogenic substrate. Although the anteroposterior diameter is the most common parameter in clinical practice, it is not a precise indicator of the left atrium’s size due to asymmetric geometry and dilatation during remodeling [22]. Atrial fractions are an early indicator of atrial dysfunction in relation to volumes and diameters, making them suitable for risk stratification [23].

Our study highlights the significant predictive ability of LAEF, LAKE, RATEF, and RAAEF, which exhibited strong discriminatory power, with an area under the curve (AUC) exceeding 0.89. These parameters offer a refined approach to preoperative risk stratification, identifying patients at the highest risk for POAF development. LAEF, a marker of left atrial pump function, was significantly reduced in POAF patients (HR: 0.030–0.167, χ^2^ = 71.842, *p* < 0.001). LAKE, which quantifies the contractile energy output of the left atrium, was also significantly reduced in POAF patients (HR: 0.298–0.563, χ^2^ = 70.082, *p* < 0.001). LAKE values are associated with the clinical worsening of heart failure. LAEF and LAKE are independent predictors of recurrent AF electroconversion and catheter ablation [24,25]. Prior studies have shown that reduced left atrial strain and impaired contractility are associated with POAF, further supporting the role of left atrial mechanics in atrial fibrillation risk [26]. RATEF was significantly lower in POAF patients (χ^2^ = 65.709, *p* < 0.001), emphasizing the importance of right atrial contractile function in maintaining sinus rhythm. Emerging evidence suggests that right atrial dysfunction is as equally critical as left atrial dysfunction in atrial fibrillation risk [6]. RAAEF was also significantly reduced (χ^2^ = 66.80, *p* < 0.001), reinforcing the role of impaired right atrial contractility in POAF development. Studies have indicated that reduced right atrial function is associated with an increased risk of postoperative arrhythmias [27].

Univariate analysis confirmed that LAEF (HR: 0.030–0.167, χ^2^ = 71.842, *p* < 0.001), LAKE (HR: 0.298–0.563, χ^2^ = 70.082, *p* < 0.001), RATEF (χ^2^ = 65.709, *p* < 0.001), and RAAEF (χ^2^ = 66.80, *p* < 0.001) exhibited a strong discriminatory ability, with an area under the curve (AUC) exceeding 0.89 for each parameter. The strength of these univariate results highlights the robust predictive value of atrial function parameters in the development of POAF. The fact that each of these echocardiographic markers demonstrated such high predictive accuracy confirms their pathophysiological relevance and clinical value in the early identification of patients at risk for POAF [10]. Importantly, although LAEF, RATEF, and RAAEF lost statistical significance in the multivariable model after adjusting for clinical covariates, their strong association with POAF in the univariate analysis suggests that they reflect key aspects of atrial function and remodeling that are partly mediated by other clinical factors. This finding underscores the importance of echocardiographic assessment as a tool for early risk stratification. The high AUC values observed for individual echocardiographic parameters in univariate analysis suggest that left and right atrial functions independently contribute to POAF risk and should be incorporated into clinical decision-making models. Even when their effect is reduced in the presence of other clinical variables, these echocardiographic markers remain valuable indicators of underlying atrial vulnerability.

Multivariable Cox analysis confirmed that LAKE (HR = 0.27, 95% CI 0.16–0.46, *p* < 0.001), hypertension (HR = 11.87, 95% CI 1.19–118.25, *p* = 0.035), left ventricular ejection fraction (EF) (HR = 1.08, 95% CI 1.01–1.15, *p* = 0.020), and peripheral vascular disease (PVB) (HR = 40.28, 95% CI 3.81–425.80, *p* = 0.002) were independent predictors of POAF after adjusting for clinical and echocardiographic variables. Importantly, the fact that LAKE remained a significant predictor after correction for clinical factors highlights the intrinsic role of left atrial function in arrhythmogenesis. LAKE represents the contractile energy output of the left atrium, and its reduction reflects impaired left atrial function and increased vulnerability to arrhythmias [24]. Interestingly, while LAEF, RATEF, and RAAEF demonstrated a strong predictive value in the univariate analysis, they lost statistical significance in the multivariable model after adjusting for clinical factors. This suggests that their predictive value may be partially mediated through clinical variables such as left ventricular function and vascular comorbidities. This aligns with previous reports showing that left atrial and right atrial mechanics are influenced by systemic hemodynamic load and ventricular function [17]. The interplay between atrial function and systemic factors, such as hypertension and peripheral vascular disease, appears to be a critical determinant of POAF development. Identifying hypertension and peripheral vascular disease as independent predictors of POAF reinforces the pathophysiological connection between systemic vascular dysfunction and atrial remodeling. Hypertension contributes to increased left atrial pressure, left ventricular diastolic dysfunction, and atrial fibrosis, creating an arrhythmogenic substrate [5]. Similarly, peripheral vascular disease reflects a state of chronic vascular inflammation and endothelial dysfunction, which may exacerbate atrial fibrosis and electrical instability.

Our findings suggest that incorporating LAEF, LAKE, RATEF, and RAAEF into preoperative evaluations could enhance risk stratification and improve early intervention strategies for POAF prevention. Preoperative evaluation of atrial functions can be a simple, non-invasive, clinically applicable, cost-effective, and highly reproducible method in POAF risk assessment, with minimal influence from translation, tethering, and image quality. These echocardiographic markers provide detailed insights into atrial function, conduction, and mechanics that traditional risk models do not fully capture. Current models incorporating clinical factors like age and COPD, such as the POAF score, may be significantly improved by integrating these novel echocardiographic predictors [28].

Additionally, our study reinforces the importance of preoperative interventions, including aggressive smoking cessation, optimized medical therapy, and closer postoperative monitoring for high-risk individuals. Further research should explore the impact of preoperative atrial conditioning strategies and hemodynamic optimization on POAF incidence.

While our study provides compelling evidence, certain limitations must be acknowledged. First, this was a single-center study, necessitating external validation in multicenter cohorts. Second, additional biomarkers of atrial fibrosis, inflammation, and hemodynamic load were not assessed but may further refine POAF risk prediction. Finally, long-term follow-up was not conducted, and future studies should evaluate whether these parameters predict late-onset atrial fibrillation beyond the early postoperative period.

## 5. Conclusions

Our study identifies LAEF, LAKE, RATEF, and RAAEF as robust echocardiographic predictors of POAF, highlighting the importance of atrial mechanics, conduction time, and chamber remodeling in arrhythmogenesis. The univariable analysis demonstrated that each of these parameters exhibited a strong predictive value, with an AUC exceeding 0.89, confirming their clinical relevance in identifying high-risk patients. Although some echocardiographic parameters lost statistical significance in the multivariable model, their strong association with POAF in univariable analysis underscores their pathophysiological importance and role in atrial dysfunction. Multivariable Cox analysis confirmed that LAKE, hypertension, left ventricular ejection fraction, and peripheral vascular disease are independent predictors of POAF, reinforcing the complex interplay between atrial function and systemic clinical factors. The fact that LAKE remained significant after adjusting for clinical covariates highlights the intrinsic contribution of left atrial function to arrhythmogenesis. These findings suggest that incorporating echocardiographic parameters such as LAEF, LAKE, RATEF, and RAAEF into preoperative screening protocols may significantly improve risk stratification and guide early interventions. Combining echocardiographic markers with established clinical risk factors could more precisely identify high-risk patients and targeted preventive strategies, including optimized medical therapy, anticoagulation, and enhanced postoperative monitoring. Further research should validate these findings in larger, diverse patient populations and explore their potential role in long-term atrial fibrillation prevention strategies. Investigating the mechanistic relationship between echocardiographic parameters and POAF pathophysiology may provide additional insights into targeted interventions and personalized treatment approaches.

## Figures and Tables

**Table 1:** Echocardiographic parameters of left and right atrial functions.

Parameters	Explanation
Left and right atrial kinetic energy (LAKE; RAKE)	Kinetic energy = ½ × stroke volume of each atrium × density of the blood × A − wave velocity. Stroke volume is calculated as the difference between pre-atrial contraction volume and the minimal volume of the corresponding atrium. For blood density, the accepted value is 1.06 g/cm^3^.
Left and right atrial ejection force (LAEF; RAEF)	Ejection force of each atrium is calculated as follows: ejection force = 0.53 × annular orifice area of corresponding atrioventricular valve × (A − wave velocity)^2^.
Left and right atrial active emptying fractions (LAAEF; RAAEF).	Active emptying fraction of each atrium is calculated as the difference between pre-atrial contraction volume and minimal volume divided by the pre-atrial contraction volume of the corresponding atrium.
Left and right atrial passive emptying fractions (LAPEF; RAPEF)	Passive emptying fraction of each atrium is calculated as the difference between maximal volume and pre-atrial contraction volume divided by the maximal volume of the corresponding atrium.
Left and right atrial total emptying fractions (LATEF; RATEF)	Total emptying fraction of each atrium is calculated as the difference between maximal volume and minimal volume divided by the maximal volume of the corresponding atrium.
Total atrial conduction time (TACT)	TACT = time from P-wave onset (ECG) to the peak of A-wave (TDI-derived atrial contraction wave) at the lateral mitral annulus.

TDI, tissue Doppler imaging; ECG, electrocardiogram.

**Table 2:** Demographic, clinical, and echocardiographic characteristics and occurrence of POAF.

Variable	With	Without	*p*
Age (years)	68.70 ± 7.64	63.86 ± 7.91	0.001
Gender (male)	36 (76.6%)	63 (75.0%)	1.000
Weight (kg)	80.94 ± 13.68	80.40 ± 13.92	0.829
Height (cm)	168.28 ± 8.57	167.75 ± 8.92	0.740
BSA (m^2^)	1.94 ± 0.19	1.92 ± 0.20	0.607
BMI (kg/m^2^)	28.57 ± 4.35	28.60 ± 4.70	0.973
HATCH score	1.79 ± 0.88	1.56 ± 1.09	0.197
CHA_2_DS_2_-VASc score	2.91 ± 1.02	2.56 ± 1.21	0.076
POAF score	2.13 ± 1.15	1.54 ± 1.10	0.005
AFRI	2.02 ± 0.94	1.74 ± 0.78	0.084
P (V1 lead) (mV)	−0.03 ± 0.07	−0.04 ± 0.09	0.356
P (avR lead) (mV)	−0.04 ± 0.12	−0.06 ± 0.12	0.437
P axis (°)	41.81 ± 32.26	44.75 ± 36.22	0.633
PR(Q) (ms)	174.28 ± 22.23	164.23 ± 27.89	0.026
Stable angina	0.17 ± 0.38	0.22 ± 0.41	0.517
Unstable angina	28 (59.57%)	41 (48.81%)	0.32
LMCA stenosis	14 (29.79%)	19 (22.62%)	0.49
Prior myocardial infarction	35 (74.47%)	55 (65.48%)	0.39
Prior PCI	12 (25.53%)	13 (15.48%)	0.24
Time from cardiac event (months)	22.74 ± 61.67	14.76 ± 46.04	0.441
Hypertension	45 (95.74%)	77 (91.67%)	0.339
Diabetes mellitus (OAD)	12 (25.53%)	27 (32.14%)	0.55
DMID	8 (17.02%)	7 (8.33%)	0.23
Hyperlipidemia	26 (55.32%)	50 (59.52%)	0.78
Smoking	26 (55.32%)	28 (33.33%)	0.02
Family history of cardiovascular diseases	33 (70.21%)	59 (70.24%)	0.40
Chronic kidney disease	4 (8.51%)	5 (5.95%)	0.85
Malignancy	1 (2.13%)	4 (4.76%)	1.00
Hypothyroidism	4 (8.51%)	4 (4.76%)	0.78
COPD	15 (31.91%)	10 (11.9%)	0.63
Peripheral vascular disease	8 (17.02%)	6 (7.14%)	0.01
Carotid stenosis	6 (12.77%)	10 (11.9%)	0.14
ARB	0.02 ± 0.15	0.10 ± 0.30	1.00
Beta-blocker dose (mg)	8.16 ± 17.53	3.05 ± 2.40	0.052
ACE inhibitor	34 (72.34%)	55 (65.48%)	0.54
Calcium antagonist	16 (34.04%)	29 (34.52%)	1.00
Statin	37 (78.72%)	62 (73.81%)	0.68
Nitroglycerin	27 (57.45%)	33 (39.29%)	0.07
SGLT 2 inhibitors	9 (19.15%)	19 (22.62%)	0.81
ASA	41 (87.23%)	70 (83.33%)	0.73
P2Y12 inhibitors	18 (38.3%)	39 (46.43%)	0.47
Oral anticoagulants	2 (4.26%)	3 (3.57%)	1.00
Amiodarone	4 (8.51%)	7 (8.33%)	1.00
Trimetazidine	15 (31.91%)	29 (34.52%)	0.91
Loop diuretic	11 (23.4%)	21 (25.0%)	1.00
Thiazide diuretics	10 (21.28%)	13 (15.48%)	0.55
Aldosterone receptor antagonists	15 (31.91%)	20 (23.81%)	0.42
Levosimendan	8 (17.02%)	8 (9.52%)	0.33

POAF, postoperative atrial fibrillation; BSA, body surface area; BMI, body mass index; HATCH, hypertension, age ≥ 75, stroke, chronic obstructive pulmonary disease, and heart failure; CHA2DS2-VASc, congestive heart failure, hypertension, age ≥ 75 (2 points), diabetes mellitus, stroke (2 points), vascular disease, age 65–74, and sex category (female); AFRI, atrial fibrillation risk index; LMCA, left main coronary artery; PCI, percutaneous coronary intervention; OAD, oral antidiabetic drugs; DMID, diabetes mellitus insulin-dependent; COPD, chronic obstructive pulmonary disease; ARB, angiotensin receptor blockers; ACE, angiotensin-converting enzyme; SGLT 2, sodium-glucose cotransporter 2; ASA, acetylsalicylic acid.

**Table 3:** Echocardiographic parameters and occurrence of POAF.

Variable	With	Without	*p*
maxLAV (mL)	75.65 ± 14.79	68.86 ± 16.05	0.0183
maxLAVi (mL/m^2^)	39.09 ± 7.26	36.08 ± 9.06	0.0439
minLAV(mL)	54.48 ± 13.05	39.62 ± 15.81	<0.001
minLAVi (mL/m^2^)	28.19 ± 6.52	20.73 ± 8.55	<0.001
pacLAV (mL)	60.24 ± 13.83	52.30 ± 15.12	0.0035
pacLAVi (mL/m^2^)	31.17 ± 6.91	27.37 ± 8.20	0.0067
MI E-wave (cm/s)	0.59 ± 0.15	0.81 ± 0.23	<0.001
MI A-wave (cm/s)	0.42 ± 0.25	0.84 ± 0.20	<0.001
MI E/A ratio	1.78 ± 0.69	1.00 ± 0.36	<0.001
MAOA (cm^2^)	4.07 ± 0.12	4.63 ± 0.21	<0.001
TACT (ms)	141.93 ± 15.23	103.59 ± 7.73	<0.001
maxRAV (mL)	58.89 ± 10.61	46.03 ± 11.87	<0.001
maxRAVi (mL/m^2^)	30.52 ± 5.31	24.03 ± 6.21	<0.001
minRAV (mL)	42.48 ± 10.26	23.94 ± 7.06	<0.001
minRAVi (mL/m^2^)	22.00 ± 5.12	12.50 ± 3.71	<0.001
pacRAV (mL)	47.39 ± 9.88	33.41 ± 8.29	<0.001
pacRAVi (mL/m^2^)	24.57 ± 4.98	17.48 ± 4.57	<0.001
TAOA (cm^2^)	7.19 ± 0.31	8.13 ± 0.34	<0.001
MAPSE (mm)	13.41 ± 5.08	14.75 ± 3.61	0.12
TAPSE (mm)	16.78 ± 4.17	20.09 ± 3.09	<0.001
TI E-wave(cm/s)	0.51 ± 0.08	0.54 ± 0.15	0.15
TI A-wave (cm/s)	0.31 ± 0.15	0.47 ± 0.08	<0.001
TI E/A ratio	1.91 ± 0.64	1.17 ± 0.30	<0.001
LATEV (mL)	21.17 ± 8.20	29.24 ± 6.60	<0.001
LATEVi (mL/m^2^)	10.68 ± 4.30	14.98 ± 4.38	<0.001
LATEF (%)	0.28 ± 0.10	0.44 ± 0.11	<0.001
LAPEV (mL)	15.41 ± 7.63	16.56 ± 6.79	0.40
LAPEVi (mL/m^2^)	7.76 ± 3.89	8.50 ± 4.09	0.31
LAPEF (%)	0.20 ± 0.09	0.24 ± 0.09	0.02
LAAEV (mL)	5.76 ± 2.09	12.68 ± 5.83	<0.001
LAAEVi (mL/m^2^)	2.92 ± 1.15	6.25 ± 3.43	<0.001
LAAEF (%)	0.10 ± 0.03	0.26 ± 0.12	<0.001
LAEF (kdyne)	0.51 ± 0.65	1.85 ± 0.94	<0.001
LAKE (kdyne·cm)	0.91 ± 1.36	5.13 ± 3.40	<0.001
RATEV (mL)	16.41 ± 5.28	22.09 ± 8.96	<0.001
RATEVi (mL/m^2^)	8.34 ± 2.96	10.84 ± 5.33	<0.001
RATEF (%)	0.28 ± 0.09	0.47 ± 0.11	<0.001
RAPEV (mL)	11.50 ± 4.82	12.62 ± 7.72	0.32
RAPEVi (mL/m^2^)	5.83 ± 2.55	6.16 ± 4.16	0.57
RAPEF (%)	0.20 ± 0.08	0.26 ± 0.10	<0.001
RAAEV (mL)	4.91 ± 1.03	9.47 ± 4.82	<0.001
RAAEVi (mL/m^2^)	2.51 ± 0.72	4.68 ± 2.79	<0.001
RAAEF (%)	0.11 ± 0.05	0.28 ± 0.12	<0.001
RAEF (kdyne)	0.44 ± 0.47	0.84 ± 0.34	<0.001
RAKE (kdyne.cm)	0.32 ± 0.38	1.11 ± 0.63	<0.001

POAF, postoperative atrial fibrillation; maxLAV, maximal left atrial volume; maxLAVi, maximal left atrial volume index; minLAV, minimal left atrial volume; minLAVi, minimal left atrial volume index; pacLAV, pre-atrial contraction left atrial volume; pacLAVi, pre-atrial contraction left atrial volume index; MI, mitral inflow; MAOA, mitral annular orifice area; TACT, total atrial conduction time; maxRAV, maximal right atrial volume; maxRAVi, maximal right atrial volume index; minRAV, minimal right atrial volume; minRAVi, minimal right atrial volume index; pacRAV, pre-atrial contraction left atrial volume; pacRAVi, pre-atrial contraction left atrial volume index; TAOA, tricuspid annular orifice area; MAPSE, mitral annular plane systolic excursion; TAPSE, tricuspid annular plane systolic excursion; TI, tricuspid inflow; LATEV, left atrial total emptying volume; LATEVi, left atrial total emptying volume index; LATEF, left atrial total emptying fraction; LAPEV, left atrial passive emptying volume; LAPEVi, left atrial passive emptying volume index; LAPEF, left atrial passive emptying fraction; LAAEV, left atrial active emptying volume; LAAEVi, left atrial active emptying volume index; LAAEF, left atrial active emptying fraction; LAEF, left atrial ejection force; LAKE, left atrial kinetic energy; RATEV, right atrial total emptying volume; RATEVi, right atrial total emptying volume index; RATEF, right atrial total emptying fraction; RAPEV, right atrial passive emptying volume; RAPEVi, right atrial passive emptying volume index; RAPEF, right atrial passive emptying fraction; RAAEV, right atrial active emptying volume; RAAEVi, right atrial active emptying volume index; RAAEF, right atrial active emptying fraction; RAEF, right atrial ejection force; RAKE, right atrial kinetic energy.

## Data Availability

The data presented in this study are available upon request from the corresponding author. The data are not publicly available due to data privacy laws.
